# Do Author-Suggested Reviewers Rate Submissions More Favorably than Editor-Suggested Reviewers? A Study on *Atmospheric Chemistry and Physics*

**DOI:** 10.1371/journal.pone.0013345

**Published:** 2010-10-14

**Authors:** Lutz Bornmann, Hans-Dieter Daniel

**Affiliations:** 1 Office of Research Analysis and Foresight, Max Planck Society, Munich, Germany; 2 Professorship for Social Psychology and Research on Higher Education, ETH Zurich, Zurich, Switzerland; 3 Evaluation Office, University of Zurich, Zurich, Switzerland; Cuban Neuroscience Center, Cuba

## Abstract

**Background:**

Ratings in journal peer review can be affected by sources of bias. The bias variable investigated here was the information on whether authors had suggested a possible reviewer for their manuscript, and whether the editor had taken up that suggestion or had chosen a reviewer that had not been suggested by the authors. Studies have shown that author-suggested reviewers rate manuscripts more favorably than editor-suggested reviewers do.

**Methodology/Principal Findings:**

Reviewers' ratings on three evaluation criteria and the reviewers' final publication recommendations were available for 552 manuscripts (in total 1145 reviews) that were submitted to *Atmospheric Chemistry and Physics*, an interactive open access journal using public peer review (authors' and reviewers' comments are publicly exchanged). Public peer review is supposed to bring a new openness to the reviewing process that will enhance its objectivity. In the statistical analysis the quality of a manuscript was controlled for to prevent favorable reviewers' ratings from being attributable to quality instead of to the bias variable.

**Conclusions/Significance:**

Our results agree with those from other studies that editor-suggested reviewers rated manuscripts between 30% and 42% less favorably than author-suggested reviewers. Against this backdrop journal editors should consider either doing without the use of author-suggested reviewers or, if they are used, bringing in more than one editor-suggested reviewer for the review process (so that the review by author-suggested reviewers can be put in perspective).

## Introduction

In the research on journal peer review, there are said to be biases, if – independently of the quality of submitted manuscripts – attributes of the reviewers (such as the nomination of a reviewer by the author or the editor) are correlated statistically with the reviewers' ratings [1]. Arkes [2] defines bias “as any systematic effect on ratings unrelated to the true quality of the object being rated. Thus, bias consists of effects that reduce the validity of ratings through contamination, but not random error” (p. 378). According to Jayasinghe [3] “a random error is an ‘unexplained’ error whereas systematic bias such as leniency/harshness of reviewers … can be explained or statistically controlled” (p. 35).

Reviewers for a manuscript can be selected by editors (1) on the basis of their personal knowledge and familiarity from past experience, (2) from a database of previous reviewers cross-referenced by name and specialty, (3) from references listed in the manuscript, and (4) based on suggestions made by the authors of the manuscript [4]. For Tonks [5], an assistant editor at the *British Medical Journal* (BMJ), the selection of author-suggested reviewers (R_a_) “could improve the quality of peer review in two important ways. Firstly, authors are often better placed than editors to know whom to approach for a considered, balanced, and credible opinion in their field of research. The best reviewers are not those with the most experience or eminence and may be unknown to anyone outside the subject. This is a particular problem for editors of general journals, who review manuscripts from a wide range of disciplines. Secondly, nominated reviewers will enrich the BMJs database, keeping us in touch with young active researchers and giving us a broader population of reviewers.”

According to the “Ethical Guidelines for Publication in Journals and Reviews” of the European Association for Chemical and Molecular Sciences [6], editors have the responsibility “to consider the use of an author's suggested reviewers for his/her submitted manuscript, but to ensure that the suggestions do not lead to a positive bias.” R_a_ may be biased in favor of the authors [7]. The danger with R_a_ is that “they can be the authors' best friends” [8] (p. 15). It is feared that through the use of R_a_ in addition to editor-suggested reviewers (R_e_) (meaning reviewers selected by the editor not on the basis of a suggestion by the author), the one (R_a_) rates a manuscript systematically more leniently than the other (R_e_). (We assume this leniency effect, although an R_e_ is not necessarily unknown to the authors.)

A number of studies of different journals showed that this fear is justified. A study by Schroter, Tite, Hutchings, and Black [9] on the peer review process at 10 biomedical journals found that R_a_ “tended to make more favorable recommendations for publication” (p. 314) than R_e_ [10]. Similar findings were reported by Scharschmidt, Deamicis, Bacchetti, and Held [11] for the *Journal of Clinical Investigation*, Earnshaw and Farndon [12] for the *British Journal of Surgery*, Goldsmith, Blalock, Bobkova, and Hall [13] for the *Journal of Investigative Dermatology*, Wager, Parkin, and Tamber [14] for medical journals in the BMC (BioMed Central) series, Rivara, Cummings, Ringold, Bergman, Joffe, and Christakis [15] for a pediatric journal, and Bornmann and Daniel [16] for *Angewandte Chemie International Edition* (AC-IE). In addition, Jayasinghe, Marsh and Bond [17] found similar results in the area of grant peer review.

In this study we aim to test whether there is a potential source of bias in the manuscript reviewing in public peer review at an interactive open access journal, *Atmospheric Chemistry and Physics* (ACP), through the use of R_a_ and R_e_. Using modern information technology, in particular the Internet, the ACP and other interactive open access journals have now become established in science that work with a “new” system of public peer review [18], [19]. Compared to the traditional system, the new system of peer review in an electronic environment is seen to have the following advantages, among others: (1) submitted manuscripts are immediately published as “discussion papers” on the journal's website, (2) reviewers' comments on the quality of the content of the manuscript and authors' replies to the reviewers' critical comments are publicly exchanged, and (3) reviewers' arguments are publicly heard, and, if comments are openly signed, reviewers can also claim authorship for their contributions [20].

Even if all studies so far have found that R_a_ rate manuscripts systematically more favorably than R_e_, it would be expected that public peer review at ACP does not show this effect. (With the exception of Wager, Parkin, and Tamber [14], the aforementioned studies conducted up to now examined traditional peer review.) Public peer review is supposed to bring a new openness to the reviewing process that will enhance its objectivity [21]. Publishing reviews is supposed to lead to reviewers using argumentation and judging solely on the basis of scientific criteria, so that the reviewer's ratings will not be influenced by potential sources of bias. We investigated the extent to which this expectation can be confirmed, taking the example of ACP.

## Methods

### Manuscript review at ACP

ACP was launched in September 2001. It is produced and published by the European Geosciences Union (EGU) (http://www.egu.eu) and Copernicus Publications (http://publications.copernicus.org/). ACP is freely accessible via the Internet (www.atmos-chem-phys.org). It has the second highest annual Journal Impact Factor (JIF) (provided by Thomson Reuters, Philadelphia, PA, USA) in the category “Meteorology & Atmospheric Sciences” (at 4.881 in the 2009 Journal Citation Reports, Science Edition). ACP has a two-stage publication process [20], [22] that is described on the ACP website as follows: In the first stage, manuscripts that pass a rapid pre-screening process (access review) are immediately published as “discussion papers” on the journal's website (by doing this, they are published in *Atmospheric Chemistry and Physics Discussions*, ACPD). These discussion papers are then made available for “interactive public discussion,” during which the comments of reviewers (usually, reviewers that already conducted the access review), additional comments by other interested members of the scientific community, and the authors' replies are published alongside the discussion paper. The reviewers can be R_a_ or R_e_.

During the discussion phase, the designated reviewers are asked to answer to the following questions according to the ACP's principal evaluation criteria (see http://www.atmospheric-chemistry-and-physics.net/review/ms_evaluation_criteria.html, from which the following information is taken): (1) scientific significance (“Does the manuscript represent a substantial contribution to scientific progress within the scope of ACP (substantial new concepts, ideas, methods, or data?”), (2) scientific quality (“Are the scientific approach and applied methods valid? Are the results discussed in an appropriate and balanced way (consideration of related work, including appropriate references)?”), and (3) presentation quality (“Are the scientific results and conclusions presented in a clear, concise, and well-structured way (number and quality of figures/tables, appropriate use of English language)?”). The response categories for the three questions are: (1) excellent, (2) good, (3) fair, and (4) poor. In addition to the principal evaluation criteria, the reviewers are asked to give a final publication recommendation: “Do you recommend acceptance of the manuscript?” Here, the response categories are: (1) yes, without alterations, (2) yes, after minor alterations, (3) yes, after major alterations, and (4) no. Besides giving the formal ratings to the four questions, the reviewers also have the opportunity to write a commentary.

The ratings are submitted in parallel to the commentaries, but they are not open, because they are meant to support the editorial decision rather than the scientific discussion. This policy was introduced in 2001. According to the experiences and the philosophy of ACP's chief-executive editor Ulrich Pöschl, prescribed publication of formal ratings is likely to do more harm than good (e.g., initiation/escalation of unnecessary controversies). Most other journals pursuing public peer review do not prescribe publication of formal ratings either, and some of them explicitly instruct reviewers not to include formal ratings in their public comments (see, e.g., http://adv-model-earth-syst.org/index.php/JAMES/about/faq). At ACP, the editors leave it up to the reviewers if they want to include ratings in their public comments, and sometimes they do (∼30%). With increasing acceptance and spread of public review it may become beneficial and appropriate to prescribe publication of formal ratings. For now, however, the ACP editors prefer a mix of open commentaries and non-public ratings for the discussion phase.

After the end of the discussion phase every author has the opportunity to submit a revised manuscript taking into account the reviewers' comments and the comments of interested members of the scientific community. Based on the revised manuscript and in view of the access peer review and interactive public discussion, the editor accepts or rejects the revised manuscript for publication in ACP. For this publication decision, further external reviewers may be asked to review the revision, if needed. In general, an editor accepts a manuscript for publication in ACP, if – similar to the “clear-cut” rule of the journal AC-IE [23] – all reviewers rate the manuscript favorably (see here http://www.atmospheric-chemistry-and-physics.net/review/ms_evaluation_criteria.html).

### Database for the present study

For the investigation of peer review at ACP we had data for 1111 manuscripts that went through the complete ACP selection process in the years 2001 to 2006 [24], [25], [26]. Of the 1111 manuscripts, 1032 (93%) manuscripts were published as discussion papers; 79 (7%) were rejected during access review for publication as discussion papers. Reviewers' ratings on the evaluation criteria and reviewers' final publication recommendations, made during the discussion phase of the reviewing process, were available for 552 (55%) of the 1008 manuscripts. This reduction in number is due to the fact that the ratings have been stored electronically by the publisher only since 2004. Of the 552 manuscripts, 16% (*n* = 87) have one review, 64% (*n* = 356) have two, 17% (*n* = 92) have three, 3% (*n* = 15) have four, and 2 manuscripts have five independent reviews. Of the total 1145 reviews, 304 (27%) were by R_a_ and 841 (73%) by R_e_.

Of the 1111 manuscripts submitted between 2001 and 2006, 958 (86%) were published in ACPD and ACP, 74 (7%) were published in ACPD but not in ACP (here, the editor rejected the revised manuscript), and 79 (7%) were published neither in ACPD nor in ACP (these manuscripts were rejected during the access review). The search for the fate of the manuscripts that were not published in ACP (*n* = 153) revealed that 38 (25%) were published as contributions in other journals. No publication information was found for 115 (75%) manuscripts, whereby 70 of the 115 manuscripts (61%) were published in ACPD. The 38 manuscripts that were published as contributions in other journals were published in 25 different journals within a time period of five years (that is, between 2005 and 2009). Six manuscripts were published in the *Journal of Geophysical Research*; three manuscripts were published in *Geophysical Research Letters*. The other 23 journals published one or two of these manuscripts each [25].

### Statistical procedures

Normally, when examining the association of a bias variable and reviewers' ratings it is impossible to establish unambiguously whether a particular group of manuscripts receives more favorable reviewers' ratings due to this variable, or if the more favorable ratings are simply a consequence of the manuscripts' scientific quality [27]. For this reason, the statistical analysis should control for the scientific quality of a manuscript [28]. Smart and Waldfogel [29] call this approach “a clean test for the existence of discrimination“ (p. 5), which in this study was realized through different statistical methods in two independent analysis steps.

To test whether R_a_ rate more leniently than R_e_, we used what is called a within-manuscript analysis as a first step. This analysis approach was proposed by Jayasinghe, Marsh, and Bond [30] for grant peer review research. They analyzed reviewers' gender as a potential source of bias in the Australian Research Council (Canberra) peer review and conducted “a within-proposal analysis based on those proposals with at least one male external reviewer and at least one female external reviewer” (p. 353). Some years later Wager, Parkin, and Tamber [14] investigated in the area of journal peer review “pairs of reviews from 100 consecutive submissions to medical journals in the BMC series (with one author-nominated and one editor-chosen reviewer and a final decision).”

At ACP between 2004 and 2006 135 of a total of 552 manuscripts (25%) were reviewed by a pair of R_a_ and R_e_. Differences in the ratings by the two reviewers of these manuscripts (related paired samples of R_a_ and R_e_) were investigated using the marginal homogeneity test [31], which generalizes the McNemar test from binary response to multinomial response. The method developed in the present release of StatXact [32] applies to ordered response. As the ACP data for the marginal homogeneity test are sparse, exact p-values were calculated.

As in the within-manuscript analysis only 135 of the 552 manuscripts could be included, an ordinal regression model (ORM) was computed as a second step to analyze ratings of R_a_ and R_e_. Using ORM, the association between several independent variables (here: suggestion of a reviewer and citations as an indicator for scientific quality) and an ordinal-scaled dependent variable (here: the reviewers' ratings) can be determined: “As with the binary regression model, the ORM is nonlinear, and the magnitude of the change in the outcome probability for a given change in one of the independent variables depends on the levels of all the independent variables” [33] (p. 183). For the analysis, the ACP data is a dataset where the assumption of independence between individual ratings of the reviewers may not hold, as the reviews are nested within manuscripts. In order to take the dependencies between individual ratings into account in the estimation of the ORMs, we used the “cluster” option in Stata [34]. Specifying this option leads to robust standard errors in the sense that the estimates provide correct standard errors in the presence of the effects of clustered data [33]. “The performance of the cluster-robust estimator is good with 50 or more clusters, or fewer if the clusters are large and balanced” [35] (p. 514). In this study we have 552 unbalanced clusters (manuscripts with one to five reviewers).

By fitting an ordinary ORM with robust standard errors for clustered data instead of fitting a variance components model (a multilevel model for ordinal responses), we were treating the within-cluster dependence as a “nuisance” and not as a phenomenon that we were interested in [36]. A Wald test by Brant [37] was performed to test the parallel regression assumption for each independent variable considered in the ORM [38]. As the test provides evidence that the assumption was violated for the variable “number of citations for a manuscript,” the variable was entered into the regression analysis as a log-transformed variable.

Out of a lack of other operationalizable indicators, it is common in research evaluation to use citation counts as an indicator for scientific quality. According to van Raan [39] citations provide “a good to even very good quantitative impression of at least one important aspect of quality, namely international impact” (p. 404). According to Lindsey [40] citations are “our most *reliable* convenient measure of quality in science – a measure that will continue to be widely used” (p. 201). In the present study we retrieved citation counts for manuscripts accepted by ACP or rejected and published elsewhere for a fixed time window of three years after the publication year. “Fixed citation windows are a standard method in bibliometric analysis, in order to give equal time spans for citation to articles published in different years, or at different times in the same year” [41] (p. 243). The citation analyses for the present study were conducted based on Chemical Abstracts (CA) (Chemical Abstracts Services, Columbus, Ohio, USA). CA is a comprehensive database of publicly disclosed research in chemistry and related sciences (see http://www.cas.org/).

As the citation counts were captured ex post – that is, after the editors' publication decisions (at ACP or another journal) – they are included in the regression models only as control variables. This means that in the analysis the interest was *not* the correlation between citation counts and reviewers' ratings but instead the correlation between the bias variable and ratings, when manuscript impact is *statistically controlled*. In statistical bias analysis this procedure is called the control variable approach [42].

## Results

Table 1 shows the minimum, maximum, mean, standard deviation, and median of the ratings by R_a_ und R_e_ on the scientific significance, scientific quality, and presentation quality of a manuscript and the final publication recommendation. Whereas the arithmetic average ratings by R_e_ are more negative on all evaluation criteria and for the final publication recommendation than the ratings by R_a_, the median ratings of the two groups do not differ on either evaluation criteria or final publication recommendation. The median ratings for the two reviewers groups are always 2. The results shown in Table 1 are not really meaningful, as they do not refer to differences between R_a_ and R_e_ on one and the same manuscript.

**Table 1:** Minimum (min), maximum (max), mean, standard deviation (sd), and median of ratings by R_a_ and R_e_ on the scientific significance, scientific quality, and presentation quality of a manuscript and final publication recommendations.

Reviewer group	Scientific significance*	Scientific quality*	Presentation quality*	Final publication recommendation$
R_a_				
n	304.00	304.00	304.00	304.00
min	1.00	1.00	1.00	1.00
max	4.00	4.00	4.00	4.00
mean	1.89	2.00	2.02	2.27
sd	0.66	0.69	0.69	0.55
median	2.00	2.00	2.00	2.00
R_e_				
n	841.00	841.00	841.00	841.00
min	1.00	1.00	1.00	1.00
max	4.00	4.00	4.00	4.00
mean	2.07	2.25	2.20	2.40
sd	0.71	0.76	0.73	0.63
median	2.00	2.00	2.00	2.00
Total				
n	1145.00	1145.00	1145.00	1145.00
min	1.00	1.00	1.00	1.00
max	4.00	4.00	4.00	4.00
mean	2.02	2.19	2.15	2.37
sd	0.70	0.75	0.72	0.61
median	2.00	2.00	2.00	2.00

*Notes*.

*Response categories: (1) excellent, (2) good, (3) fair, and (4) poor.

$ Response categories: (1) yes, without alterations, (2) yes, after minor alterations, (3) yes, after major alterations, (4) no.

Table 2 presents the results of the within-manuscript analysis. For each evaluation criterion and for the final publication recommendation the table shows the difference between the ratings of reviewers for those manuscripts (n = 135) that were each reviewed by an R_a_ and an R_e_. The table shows the number of those manuscripts (row percents) for which the ratings by R_a_ and R_e_ did not differ (column: “no difference”), the rating by R_a_ was more positive than the rating by R_e_ (column: “R_a_ is more positive than R_e_”), and the rating by R_e_ was more positive than the rating by R_a_ (column: “R_e_ is more positive than R_a_”). As the distribution of the percentage values for all evaluation criteria and for the final publication recommendation show, there are clearly more manuscripts rated more favorably by R_a_ than by R_e_ than there are manuscripts rated more favorably by R_e_ than by R_a_. For instance, 22% of the final publication recommendations made by R_a_ are more positive than those made by R_e_. There are more positive recommendations by R_e_ than by R_a_ for only 11% of the manuscripts (there is no difference between the recommendations by the two reviewer groups for 67% of the manuscripts). Hence, overall for this group of manuscripts R_a_ rated more favorably than R_e_ more frequently than vice versa. Using the marginal homogeneity test, we examined whether the ratings by R_a_ and R_e_ also differed statistically significantly. As the results of the test in Table 2 show, the difference is statistically significant only for the final publication recommendation. The differences between the ratings on the evaluation criteria are non-significant.

**Table 2:** Differences between the ratings by R_a_ and R_e_ on three evaluation criteria and on the reviewers' final publication recommendations for those manuscripts that were each reviewed by both an R_a_ and an R_e_ (n = 135).

Evaluation criteria and final publication recommendation	Difference between R_a_ and R_e_ (row percent):			Marginal homogeneity test (χ^2^)
	No difference	R_a_ is more positive than R_e_	R_e_ is more positive than R_a_	
Scientific significance§	53	29	18	1.75
Scientific quality§	47	30	23	1.54
Presentation quality§	49	31	20	1.53
Final publication recommendation$	67	22	11	1.87*

*Notes*.

§ Response categories: (1) excellent, (2) good, (3) fair, and (4) poor.

$ Response categories: (1) yes, without alterations, (2) yes, after minor alterations, (3) yes, after major alterations, (4) no.

*p<.05 (marginal homogeneity test for ordered data; one-side p-value, difference occurs in the hypothesized direction).

The differing results of the marginal homogeneity test could indicate that with the same ratings on all evaluation criteria, R_a_ tend to make a more positive final publication recommendation than R_e_. To test this hypothesis, in a further analysis we selected those manuscripts among the 135 manuscripts reviewed by both R_a_ and R_e_ that were rated the same on all evaluation criteria by both reviewers. This was the case for 18% of the manuscripts (n = 24). Table 3 shows the reviewers' ratings on the evaluation criteria and their final publication recommendations for the 24 manuscripts. Whereas the final publication recommendations by both reviewers were the same for 21 manuscripts, for 3 manuscripts the final publication recommendations by R_a_ were more favorable than the recommendations by R_e_. No manuscript received a more favorable final publication recommendation by R_e_ than by R_a_.

**Table 3:** Final publication recommendation by R_a_ and R_e_ for those manuscripts, for which an R_a_ and an R_e_ gave identical ratings on three evaluation criteria (n = 24).

Evaluation criteria§			Final publication recommendation$	
Scientific significance	Scientific quality	Presentation quality	R_e_	R_a_
1	1	1	Minor	**Accept**
1	1	1	Minor	Minor
1	1	2	Minor	Minor
1	2	2	Minor	Minor
2	1	1	Minor	Minor
2	2	2	Major	Minor
2	2	2	Minor	Minor
2	2	2	Minor	Minor
2	2	2	Minor	Minor
2	2	2	Minor	Minor
2	2	2	Minor	Minor
2	2	2	Minor	Minor
2	2	2	Minor	Minor
2	2	2	Minor	Minor
2	2	2	Minor	Minor
2	2	2	Major	**Minor**
2	2	3	Minor	Minor
2	2	3	Minor	Minor
2	3	3	Major	**Minor**
2	3	3	Major	Major
2	3	3	Major	Major
3	2	3	Minor	Minor
3	3	2	Major	Major
3	3	3	Major	Major

In the table, three final publication recommendations where R_a_ made a more favorable recommendation than R_e_ are shown in **bold**.

*Notes*.

§ Response categories: (1) excellent, (2) good, (3) fair, and (4) poor.

$ Response categories: (Accept) yes, without alterations, (Minor) yes, after minor alterations, (Major) yes, after major alterations.

In closing, we tested differences between the ratings by R_a_ and R_e_ using ORMs. An ORM was computed for each evaluation criterion and the final publication recommendation. Table 4 presents a description of the dependent and independent variables that were included in the total of four ORMs. The independent variables are “Author-suggested reviewer” (R_a_ or R_e_) and the log-transformed citation counts. Table 5 shows the results of the ORMs. For all ORMs the variable “Author-suggested reviewer” has a statistically significant effect in the expected direction: If the review is by R_a_, the ratings on all criteria as well as the final publication recommendation are statistically significantly more favorable than the ratings, if the review is by R_e_ – independently of the quality of the reviewed manuscript (measured ex-post using citation counts). To be able to assess the size of the effect of the variable “Author-suggested reviewer” on the ratings, after the ORMs we computed percent changes in expected ratings for a unit increase (from rating by R_e_ to rating by R_a_) [33]. As the results in Table 5 show, in reviews by R_e_ ratings can be expected that are between 30% and 42% less favorable than the ratings by R_a_.

**Table 4:** Description of the dependent and independent variables included in the ORM.

Variable	Range of values	Arithmetic mean
Dependent variable		
Scientific significance	1→4	2.02
Scientific quality	1→4	2.19
Presentation quality	1→4	2.15
Final publication recommendation	1→4	2.37
Independent variables		
Author-suggested reviewer (1 = R_a_, 0 = R_e_)	0→1	0.27
Citation counts for the first three years after the publication year (measured ex post, log-transformed)	0→4.56	1.80

**Table 5:** Results of the ORM predicting reviewers' ratings for three evaluation criteria and the final publication recommendation.

Independent variable	Scientific significance§	Scientific quality§	Presentation quality§	Final publication recommendation$
Fixed part: Maximum likelihood estimates				
Citation counts for the first three years after the	−0.434***	−0.434***	−0.278***	−0.368***
publication year (measured ex post, log-transformed)	(−5.81)	(−7.04)	(−4.04)	(−4.86)
Author-suggested reviewer (1 = R_a_, 0 = R_e_)	−0.408**	−0.552***	−0.406**	−0.350*
	(−3.08)	(−4.30)	(−3.10)	(−2.57)
Fixed part: Thresholds				
К_1_	−2.337***	−2.706***	−2.228***	−4.383***
	(−12.93)	(−17.38)	(−13.41)	(−17.16)
К_2_	0.567***	0.00570	0.292*	−0.125
	(3.68)	(0.05)	(2.02)	(−0.82)
К_3_	2.740***	2.142***	3.026***	2.385***
	(11.78)	(12.60)	(12.84)	(11.72)
*n_reviews_*	1145	1145	1145	1145
*n_manuscripts_*	552	552	552	552
Reviews per	min = 1	min = 1	min = 1	min = 1
manuscript	mean = 2.1	mean = 2.1	mean = 2.1	mean = 2.1
(cluster)	max = 5	max = 5	max = 5	max = 5
Change in expected rating for a unit increase in “Author-suggested reviewer”	-34%	-42%	-33%	-30%

*Notes*.

*p<0.05,

**p<0.01,

***p<0.001

§ Response categories: (1) excellent, (2) good, (3) fair, and (4) poor.

$ Response categories: (1) yes, without alterations, (2) yes, after minor alterations, (3) yes, after major alterations, (4) no.

t statistics in parentheses.

## Discussion

Compared to most of the studies on potential sources of bias in the manuscript reviewing process published up to now, the present study used an optimized strategy with two independent analysis steps. In both steps there was a control for the scientific impact of the research reported in a manuscript in order to be able to determine – *independently of their quality* – whether manuscripts that were reviewed by R_a_ are reviewed more favorably than manuscripts that were reviewed by R_e_. The results of this study are therefore more solid than the results of most of the studies published up to now that did not control for the scientific impact of manuscripts in the evaluation.

In a first step of analysis, we used a within-manuscript approach. Even though this analysis revealed a statistically significant difference between the reviews by R_a_ and R_e_ only with regard to the final publication recommendation (and not for the evaluation criteria), there is a tendency in the dataset towards more manuscripts that R_a_ rate more favorably than R_e_ than the opposite case. In addition, with the same ratings on the evaluation criteria, R_a_ tends towards a more positive than a more negative final publication recommendation than R_e_. In a second step of analysis, an ORM was computed. This analysis showed that both for the evaluation criteria and the final publication recommendations, more positive ratings can be expected by R_a_ than by R_e_. All in all, the results for the journal ACP agree with the results of other studies (see the introduction section) and indicate that the bias variable “Author-suggested reviewer” has an effect on the reviewing process.

However, even though the results of the study indicate that there are differences between the ratings by R_a_ and R_e_, the results should be seen as only an *indication* of a potential source of bias in the ACP peer review process and *not* as proof of favoritism of certain manuscripts by R_a_. Strictly speaking, solid findings on the existence of biases in peer review processes can be produced only by experimental studies in which the research objects (such as manuscripts) are randomly assigned to a treatment and control group (such as R_a_ and R_e_) [43]. As a study of that kind would influence the review process, there is a risk of infringing the rules of good scientific practice, as pointed out by critical commentaries on the study published by Peters and Ceci [44] (see *Behavioral and Brain Sciences*, 1982, pp. 196–246, and *Behavioral and Brain Sciences*, 1985, pp. 743–747). In that study manuscripts with fictitious author names and institutional affiliations were submitted to journals for publication.

Regardless of what the results of experimental studies of that kind would be, we can probably assume that there can be no peer review system without the influence of potential sources of bias. Scientists, too, are only human: “Philosophers and sociologists agree that the notion of a truly objective disinterested ‘seeker after truth’ is incompatible with the realities of social existence. We all have personal interests and institutional values that we are bound to promote in our scientific work … It will surely defend objectivity as an ideal, impossible to realize completely in practice but always to be respected and desired” [45] (p. 754). To obtain an indication of the systematic influence of sources of bias in a peer review process, in research evaluation it is proposed that the process of peer reviewing should be studied continuously and that any evidence of bias in the process should be brought to the attention of the editor for correction and modification of the process [46], [47]. Hojat, Gonnella, and Caelleigh [48] demanded “that the journal editors conduct periodic internal and external evaluations of their journals' peer review process and outcomes” (p. 75) to assure the integrity of the process. In the most comprehensive review of research on biases in peer review, Godlee and Dickersin [49] also concluded that “journals should continue to take steps to minimize the scope for unacceptable biases, and researchers should continue to look for them” (p. 112).

If indications of the effect of sources of bias are found in a peer review process, Thorngate, Dawes, and Foddy [50] recommend the following measures “to fix the problem … One possible solution is to replace biased judges with neutral ones. Another is to train and to motivate offending judges to mend their judgmental ways. A third is to add more judges in hopes that their biases will counterbalance each other and produce a neutral group consensus. Each is worthy of brief consideration” (p. 55). This study showed, in agreement with all other studies, for the bias variable investigated that independently of the quality of a manuscript, better ratings can be expected from R_a_ than from R_e_. Many journals use precautions to avoid biased review from R_a_, e.g., by stipulating that reviewers do not work in the same institution, have never published with them, etc. If reviewers have a disqualifying conflict they should excuse themselves or not be used. However, personal relationships are harder to quantify than financial links so they are often overlooked. Journal editors should therefore consider, if R_a_ are used, bringing in more than one R_e_ for the review process so that the review by R_a_ can be put in perspective.
